# *Podophyllum hexandrum*-Mediated Survival Protection and Restoration of Other Cellular Injuries in Lethally Irradiated Mice

**DOI:** 10.1093/ecam/nep061

**Published:** 2011-02-13

**Authors:** Sanghmitra Sankhwar, Manju Lata Gupta, Vanita Gupta, Savita Verma, Krishna Avtar Suri, Memita Devi, Punita Sharma, Ehsan Ahmed Khan, M. Sarwar Alam

**Affiliations:** ^1^Division of Radiation Biosciences, Institute of Nuclear Medicine and Allied Sciences, Brig. S. K. Majumdar Marg, Timarpur, New Delhi 110054, India; ^2^Natural Products Chemistry Division, IIIM (CSIR), Canal Road, Jammu 180001, India; ^3^NMR Research Center, Institute of Nuclear Medicine and Allied Sciences, Brig. S. K. Mazumdar Marg, New Delhi 110054, India; ^4^Department of Medical Elementology and Toxicology, India; ^5^Department of Chemistry, Jamia Hamdard, Hamdard University, New Delhi 110062, India

## Abstract

This study aims at the development of a safe and effective formulation to counter the effects of lethal irradiation. The sub-fraction (G-001M), prepared from *Podophyllum hexandrum* has rendered high degree of survival (>90%) at a dose of 6 mg kg^−1^ body weight (intramuscular) in lethally irradiated mice. Therapeutic dose of G-001M, at about 20 times lower concentration than its LD_100_, has revealed a DRF of 1.62. Comet assay studies in peripheral blood leukocytes have reflected that, treatment of G-001M before irradiation has significantly reduced DNA tail length (*P* < .001) and DNA damage score (*P* < .001), as compared to radiation-only group. Spleen cell counts in irradiated animals had declined drastically at the very first day of exposure, and the fall continued till the 5th day (*P* < .001). In the treated irradiated groups, there was a steep reduction in the counts initially, but this phase did not prolong. More than 60% decline in thymocytes of irradiated group animals was registered at 5 h of irradiation when compared with controls, and the fall progressed further downwards with the similar pace till 5th day of exposure (*P* < .001). At later intervals, thymus was found fully regressed. In G-001M pre-treated irradiated groups also, thymocytes decreased till the 5th day but thereafter rejuvenated and within 30 days of treatment the values were close to normal. Current studies have explicitly indicated that, G-001M in very small doses has not only rendered high survivability in lethally irradiated mice, but also protected their cellular DNA, besides supporting fast replenishment of the immune system.

## 1. Introduction

Intensive generation of reactive oxygen species (ROS), after ionizing radiation, interferes with cellular entities, leading to lipids peroxidation, protein denaturation and formation of DNA lesions, resulting into cell death [1, 2]. To counter such situations, the development of a safe and effective radioprotector is considered important [1, 2]. Characteristically, an ideal radioprotector should be easy to administer, minimally toxic, chemically stable and preferably cost-effective [3].

After achieving a very limited success in the development of chemical radioprotector, efforts were made to evaluate the protective potential of plant based preparations, against ionizing radiations [4–7]. Several herbal supplements capable of mitigating oxidative stresses were screened for their radioprotective potential [5, 6].

*Podophyllum hexandrum* (family: Berberidaceae; common name: Himalayan Mayapple), which is used to treat various bacterial and viral infections, metabolic disorders, leukemia, lymphomas, venereal warts, rheumatism, and so forth [8–10], has also been evaluated for its radioprotective capabilities. In the last one decade, this herb available at high altitudes has been explored extensively for the development of a safe radioprotector. During initial studies, the plant was used in its crude extract form, but the therapeutic doses (200 mg kg^−1^ body weight) of the extract were high [11], and most of the studies were focused on *in vitro* [12–15] and individual organ system assays namely, spermatogenesis and Jejunal changes [16, 17], hematopoietic variations [18], mitochondrial damage [19], immune-suppression [20], radiation induced neuronal changes in post natal rats, and so forth [21]. Efforts were initiated to unravel the possible mechanism of action rendered by *P. hexandrum* for radioprotection. The anti-tumor activity of this herb was observed in mice [22]. Studies including whole body survival, toxicity, biochemical and hematopoietic parameters using intraperitoneal route of drug administration, were also published recently [23, 24]. However, it has always been a sincere endeavor of all the explorers working with *P. hexandrum* to generate the advanced and valuable data in every successive study, with targeted aim of developing a safe and effective radioprotector, particularly, to protect and repair the damages that could be inflicted during nuclear eventualities.

In this investigation, minimal dose of semipurified extract (G-001M) of *P. hexandrum* was administered intramuscularly in lethally irradiated mice, to ascertain its radioprotective efficacy. Besides, many other basic parameters like maximum tolerated dose (MTD), acute toxic doses, therapeutic time window and dose reduction factor (DRF) were evaluated. The extent of protection to immune system and to DNA including support for its repair by sub-fraction G-001M was also affirmed.

## 2. Methods

### 2.1. Collection of Plant Material and Extract Preparation

The rhizomes of *P. hexandrum* Royale collected from the high-altitude regions (>3000 m) of India, were maintained free from soil, dust, insects, fungal and other extrinsic contaminations. The material was shade dried in specially designed dust-free chambers, and crushed to obtain a homogenous fine powder. The powdered material (2.3 kg) was extracted with petroleum ether (60–80°C) in a Soxhlet for 16 h. The marc (plant material left behind after extraction with different solvents) was dried to remove residual solvent, and then, further extracted with 95% ethanol in Soxhlet for 20 h. The extract, after removal of ethanol under reduced pressure, coded as G-001M yielded 550 g of dry powder.

### 2.2. Antioxidative Potential of G-001M (*P. hexandrum* Rhizome Sub-Fraction)

The anti-oxidative activity of G-001M was evaluated in terms of reducing power, using the method of Oyaizu [25]. The different concentrations of G-001M (0.05–2.0 mg) in the final volume of 50 *μ*l were mixed with 200 *μ*l of 0.2 M phosphate buffer (pH 6.5) and 0.1% potassium ferricyanide each, and incubated at 50°C in a hot water bath for 20 min. Simultaneously, butylated hydroxy toluene (BHT), a standard antioxidant used as a control, was prepared in a similar way. After adding 250 *μ*l of 10% trichloroacetic acid (TCA), the preparation was centrifuged at 3000 rpm for 10 min. The resulting supernatant, mixed with 500 *μ*l of double distilled de-mineralized water and 100 *μ*l of 0.1% ferric chloride, was incubated at 37°C for 10 min. The developed color was measured against a blank containing phosphate buffer, potassium ferricyanide and TCA. The absorbance, an indicator of reducing power, was recorded at 700 nm, using a spectrophotometer.

### 2.3. G-001M Administration

Swiss albino strain “A” male mice of 8–10 weeks old (28 ± 2 g), fed on standard animal food and water *ad libitum*, were maintained under standard animal house conditions. The experiments were carried out following the guidelines of Institute Animal Ethical Committee (IAEC). Each experiment with six animals was repeated three to four times. Required quantities of G-001M were administered in mice through intramuscular route in a maximum volume of 0.2 ml. Dimethyl sulphoxide (10% DMSO) was used as a solvent.

### 2.4. Irradiation

The animals were exposed to gamma radiation in ^60^Co gamma chamber (Model-220, Atomic Energy of Canada Ltd.) at a dose rate of 0.47–0.40 cGy s^−1^. The mice were irradiated individually, with constant supply of fresh air. Dosimetry was carried out using Baldwin Farmer's secondary dosimeter and Fricke's chemical dosimetry method.

### 2.5. Whole Body Survival and Body Weight

The effect of variable doses of G-001M, administered prior to lethal irradiation was investigated on the whole body survival of lethally irradiated mice. The data was recorded every day, up to 30 days of post-irradiation, and the same was expressed as percent survival. Mice used for the study were divided into control, 10% DMSO treated, G-001M treated, G-001M pretreated-irradiated and irradiated only groups. The body weight of animals was also registered every alternate day. To evaluate the effective time window, animals were administered with the most effective dose of G-001M, at different time intervals prior to the whole body irradiation. The time interval of study ranged between 15 min and 4 h.

Dose reduction factor was calculated by the method of Miller and Tainter [26]

(1) $$\text{DRF}=\frac{{\text{LD}}_{50}\;\text{ of G}-001\text{M pretreated irradiated group}}{{\text{LD}}_{50}\;\text{ of irradiated group}}.$$

### 2.6. MTD and Toxic Doses (LD_50_  and LD_100_ ) of G-001M

The dose of G-001M, which did not bring any toxic manifestations/death in the experimental animals, was considered as the maximum tolerable dose (MTD). The concentrations of sub-fraction amounting to 50% and 100% mortality were known as LD_50_ and LD_100_, respectively. The animals were observed up to 30 days for their morbidity and mortality status.

### 2.7. Comet Assay

Blood, collected from mice by retro-orbital bleeding using fine glass capillaries and stored in heparinized microtubes was followed for alkaline single cell gel electrophoresis using the method of Singh et al. [27] with some modifications. In brief, 10 *μ*l of heparinized blood was added to 190 *μ*l of 0.5% low melting agarose in phosphate buffer saline (PBS) at 37°C in a microtube. The mixture was uniformly spread over fully frosted slides precoated with 200 *μ*l of 1.0% normal melting agarose in PBS and kept for gelling at 0°C for 5 min. Cell lysing was done in freshly prepared ice cold lysis solution [2.5 M sodium chloride (NaCl), 100 mM disodium ethylene diamine tetra-acetic acid (EDTA), 10 mM tris, 1% sodium lauryl sarcosine, 10% dimethyl sulphoxide (DMSO), 1% triton-X-100, pH 10] for 1 h at 4°C. Prepared slides were transferred to a horizontal electrophoretic apparatus containing fresh chilled alkaline buffer [300 mM sodium hydroxide (NaOH), and 1 mM EDTA, pH 13] for 20 min and then subjected to constant electric field of 1.5 V/cm for another 20 min. After three washes with neutralizing buffer (0.4 M tris pH 7.5), the slides were stained for 20 min with propidium iodide. Analysis of DNA damage and score was done using Jaloszynski equation [21]. At least 100 cells were measured for each treatment.

### 2.8. Splenocytes and Thymocytes Preparation

For the estimation of total splenocytes and thymocytes, the mice were divided into three groups, that is, control, irradiated and G-001M pretreated irradiated. Except control, the other two groups were further subdivided into different time intervals from 5 h to 30th day, with three animals in each subgroup. Control group had no treatment. Irradiation group was exposed to 10 Gy whole body gamma irradiation. In G-001M pre-treated and irradiated group, the formulation was administered (intramuscularly) 1 h before irradiation. Animals were sacrificed by cervical dislocation at each time interval, and their thymus and spleen were excised as a whole.

### 2.9. Isolation of Splenocytes

The splenocytes, released by cutting the spleen were suspended in 2 ml ice-cold RPM1-1640 (Gibco) medium. The suspension was gently forced through a sterilized nylon mesh (60 *μ*m) to get a single cell preparation. After centrifugation at 1000 rpm for 10 min at 4°C, the pellet was suspended in 2 ml RBC lysis buffer (0.15 M ammonium chloride, 10 mM potassium bicarbonate, 0.1 mM disodium EDTA). Supernatant was discarded after further centrifugation, and the pellet was re-suspended in culture medium. Splenocytes were counted using Neubauer's Chamber.

### 2.10. Isolation of Thymocytes

Carefully removed and gently crushed thymus in PBS was passed through 25 gauge needle to obtain single cell suspension. The isolated cells were suspended in RPMI-1640 and centrifuged at 1000 rpm for 10 min at 4°C. Pellet formed was re-suspended in standard buffered saline. Cells were counted using Neubauer's Chamber.

### 2.11. Statistical Analysis

The statistical significance between control and experimental values for whole body survival was analyzed by Kaplan-Meier survival curves and Log Rank test using SPSS Software (version 10). Finney's Probit analysis for toxicity and one way ANOVA using SPSS (version 10) software was applied for DNA damage in terms of tail length. Splenocytes and thymocytes counts were evaluated using Student's *t*-test. All the values are reported as mean ± SD. A value of *P* < .05 is considered as statistically significant.

## 3. Results

### 3.1. Chemoprofiling of G-001M by HPLC

**Figure 1:**
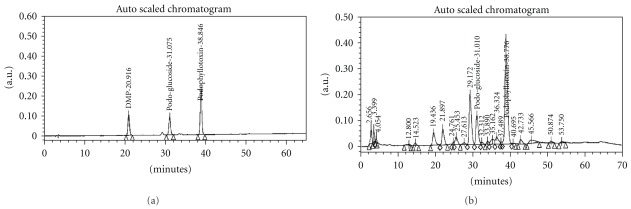
(a) HPLC chromatogram of lignans (marker). (b) HPLC chromatogram of *P. hexandrum* sub-fraction (G-001M) using methanol: water gradient over a period of 70 min: Column; RP-18 (E. Merck, 5 *μ*m, 4.0 × 250 mm); Flow rate 0.6 ml/min; UV 290 nm.

**Figure 2:**
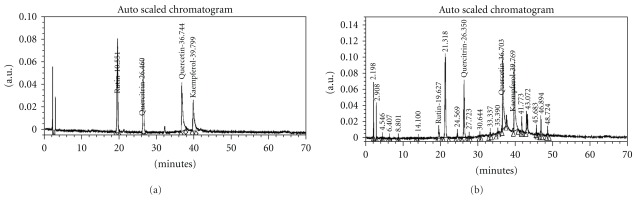
(a) HPLC chromatogram of flavonoids (markers). (b) HPLC chromatogram of *P. hexandrum* sub-fraction (G-001M) using acetonitrile containing 0.05% of TFA: water containing 0.05% of TFA gradient over a period of 70 min: Column; RP-18 (E. Merck, 5 *μ*m, 4.0 × 250 mm); Flow rate 1.0 ml/min; UV 250 nm.

HPLC Tables 1 and 2 indicated presence of lignans namely, Podophyllotoxin, Podophyllotoxin glucoside and Demethyl podophyllotoxin (Table 1, Figures 1(a) and 1(b)) and flavonoids namely, quercetin, quercitrin, rutin and kaempferol (Table 2, Figures 2(a) and 2(b)).

### 3.2. Antioxidant Potential of G-001M

**Figure 3:**
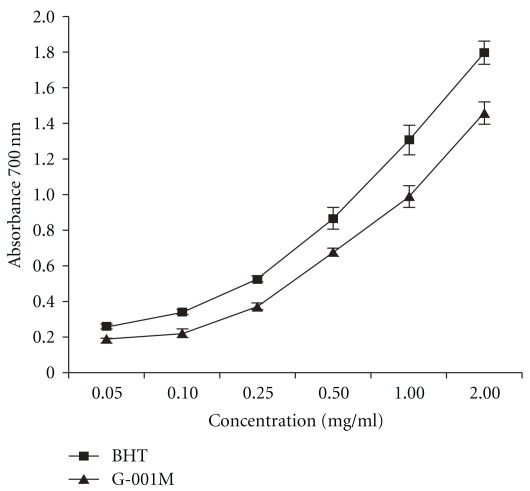
Reducing power evaluation of different concentrations of *P. hexandrum* sub-fraction (G-001M). BHT, a synthetic antioxidant was used as a standard. The absorbance at 700 nm was recorded in triplicate by spectrophotometric detection of the Fe^3+^-Fe^2+^ transformation. Experiments were repeated in triplicate. All the values are reported as mean ± SD.

Figure 3 shows the reducing potential of G-001M compared to BHT used as standard. The antioxidant activity of sub-fraction was found increased with increasing concentration of G-001M. At a particular concentration, this formulation was able to reduce most of the ferric ions (Fe^3+^) to ferrous ions (Fe^2+^). The results were comparable with the standard used in the study.

### 3.3. Maximum Tolerated Dose (MTD) and Acute Toxic Dose

**Figure 4:**
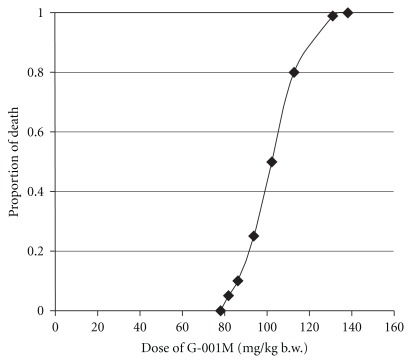
Predicted proportion of death by different doses of the *P. hexandrum* sub-fraction (G-001M) administered intra-muscularly in Swiss albino mice. The experiments were repeated thrice with each group consisting of six animals. The data observed from acute toxicity test of different levels of drugs were evaluated using Finney's Probit Analysis method.

No adverse symptoms were seen on intramuscular administration of single dose of G-001M up to 80 mg kg^−1^ body weight. After the administration of 100 mg kg^−1^ body weight of G-001M, 50% of the animals died within 72 h (Figure 4), and on single intramuscular administration of 120 mg kg^−1^ body weight of G-001M, all the animals were found dead at the same time interval. Finney's Probit analysis was used for toxicity data analysis. The predicted model on observed data indicated 78.04 mg as MTD and 102.194 mg as LD_50_, with 95% confidence interval (CI).

### 3.4. Survival Studies

**Figure 5:**
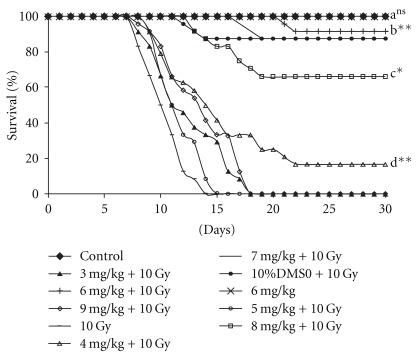
Different doses of *P. hexandrum* sub-fraction (G-001M) were administered to Swiss Albino mice (i.m.) 1 h before 10 Gy whole body irradiation. Animals were observed in terms of mortality for 30 days. Experiments were repeated four times having six animals in each group. Pooled data was analysed by Kaplan Meier survival curves. a: Normal versus 6 mg; b: 10 Gy versus 6 mg/kg + 10 Gy; c: 8 mg/kg + 10 Gy versus 6 mg/kg + 10 Gy; d: 4 mg/kg + 10 Gy versus 6 mg/kg + 10 Gy (*P < .05, **P < .001, ns, non significant).

In radiation-only group, all the animals died within 12 post-irradiation days (Figure 5). The animals administered with 10% DMSO alone, before irradiation, died with the similar death pattern as in radiation-only group. Administration of G-001M 1 h before 10 Gy whole body irradiation rendered >90% survival, as recorded up to 30 days (Figure 5). Animals in this group died their natural death at later intervals. G-001M in less than 4 mg kg^−1^ or more than 8 mg kg^−1^ body weight was found to be effective (Figure 5) but not to the same degree as when the sub-fraction was used in the range of 5–7 mg kg^−1^ body weight. The animals' overall health, including their body weight, recovered consistently after 10 days of exposure in G-001M pre-treated and irradiated group while in irradiated group, their health deteriorated by each passing day (data not shown). The most effective concentration was administered at various time gaps (from 15 min to 4 h, every second study had the gap of 30 min except in between 15 min and 30 min group where the gap was only 15 min) between G-001M administration and radiation exposure. Except 15 min and 4 h gap study, all the intervals have reflected >90% survival. A time gap of 15 min exhibited only 17% survival while 4 h interval could provide up to 66%. More than 90% of the animals were saved when the time gap between G-001M administration and whole body irradiation was in between 30 min and 3.5 h.

The survival data was analyzed using Kaplan-Meier survival curves (Figure 5) and the Log Rank test. The Log Rank statistics revealed that the survival patterns of mice which were given 6 mg of the drug, has a slightly similar pattern as that of 7 mg (*P* = .598) and 5 mg (*P* = .598) but the Kaplan-Meier curves reflected a better pattern and a narrower mean confidence interval (28.33, 30.25) with 6 mg kg^−1^ of the formulation than that of 7 mg (26.87, 30.13) and 5 mg (25.57, 30.18). It also showed higher mean survival time (29.08 ± 0.92) with 95% CI.

**Figure 6:**
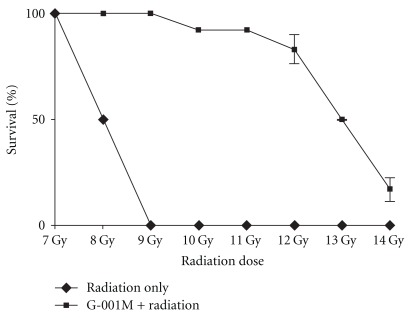
Administration (i.m.) of *P. hexandrum* sub-fraction (G-001M), 1 h prior to 7–14 Gy whole body irradiation. Survival was measured upto 30 days. DRF was estimated as the ratio of the LD_50_ value of radiation in drug treated group to the untreated group. Experiments were repeated three times with six animals in each group and all the values are represented as mean ± SD.

Whole body survival studies against supra-lethal dose (13 Gy) of radiation gave an approximate value of DRF = 1.62 (Figure 6). Pre-administration of G-001M in 14 Gy exposed mice could save only 17% of the animals (Figure 6). In radiation only group, 50% of the animals died within 12 days after 8 Gy whole body gamma irradiation.

### 3.5. Comet Assay

**Figure 7:**
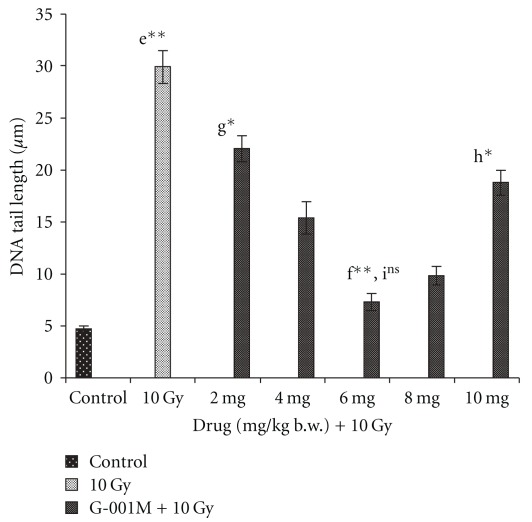
Effect of increasing concentrations of *P. hexandrum* sub-fraction (G-00IM) on DNA damage in whole body irradiated (10 Gy) mice. DNA damage was measured in terms of tail length (mean ± SD) in peripheral blood leukocytes of mice using comet assay. Experiments were done in triplicate with three animals in each group. Data points are mean values of 100 cells from each group. e: Normal versus 10 Gy; f: l0 Gy versus 6 mg + 10 Gy; g: 6 mg/kg + 10 Gy versus 2 mg/kg + 10 Gy; h: 6 mg/kg + 10 Gy versus 10 mg/kg + 10 Gy; i: Normal versus 6 mg/kg + 10 Gy (**P < .001, ns, non significant).

**Figure 8:**
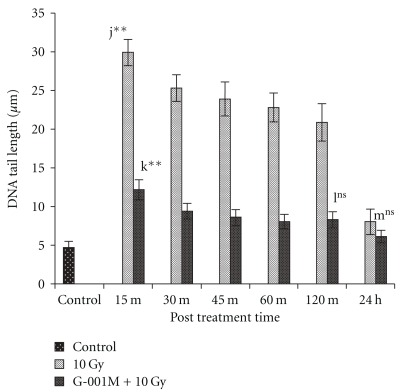
Effect *of P. hexandrum* sub-fraction (G-001M) on DNA damage in peripheral blood leucocytes of whole body irradiated (10 Gy) mice. Measurement of DNA damage was done at different time intervals using comet assay in terms of tail length (mean ± SD). Experiments were done in triplicate with three animals in each group. Data points are mean values of 100 cells from each groups. j: Normal versus 15 min 10 Gy; k: l5 min l0 Gy versus 15 min (G-001M + 10 Gy); l: 120 min 10 Gy versus l20 min (G-001M + 10 Gy); m: 24 h 10 Gy versus 24 h (G-001M + 10 Gy) (**P < .001, ns, non significant).

Whole body lethal irradiation (10 Gy) in mice infuses apparent damage to cellular DNA. The measurement of DNA damage in terms of tail length, depending on various doses of G-001M and at different time intervals, is expressed in Figures 7 and 8, respectively. The DNA damage score data has not been shown in tabular or figure form. Exposure to lethal doses of gamma radiation increased DNA tail length to 29.9 ± 1.67 *μ*m (*P* = .000, control versus 10 Gy) and DNA damage score to 97.3 (15 min post irradiation), against control values of 4.86 ± 0.79 *μ*m and 18, respectively, in peripheral blood leukocytes of irradiated mice. However, both DNA tail length and DNA damage score receded with respect to increase in sampling time. At 120 min post irradiation the tail length and DNA damage score decreased to 20.87 ± 1.64 *μ*m and 68.1, respectively. Within 24 h of study, DNA tail length (8.1 ± 0.95 *μ*m) and DNA damage score (31.5) in radiation exposed group were found close to control group (*P* = .117, ns). Pretreatment of single dose of G-001M (6 mg kg^−1^ body weight) in lethally irradiated mice extended nearly 60% protection to peripheral blood leukocytes as DNA tail length reduced to 12.2 ± 1.28 *μ*m (*P* = .000), and DNA damage score to 48.6 (15 min post irradiation). Sampling at 120 min post irradiation in pre-treated irradiated group revealed further shortening of tail length to 8.3 ± 1.04 *μ*m, and reduction in DNA damage score to 29. The similar trend continued at 24 h also, where DNA tail length shortened to 6 ± 0.78 *μ*m and DNA damage score to 23.3 (Figure 8). Concentrations of G-001M below 5 mg and more than 7 mg kg^−1^ body weight were found less efficient in exhibiting protection to leukocytic DNA, as depicted in Figure 7. Pre-administration of 10 mg kg^−1^ body weight of G-001M could decrease the DNA tail length (15 min sampling time) up to 18.8 ± 0.81 *μ*m only, as against 7.28 ± 0.52 *μ*m in 6 mg kg^−1^ body weight treated group.

### 3.6. Total Splenocytes Count

**Figure 9:**
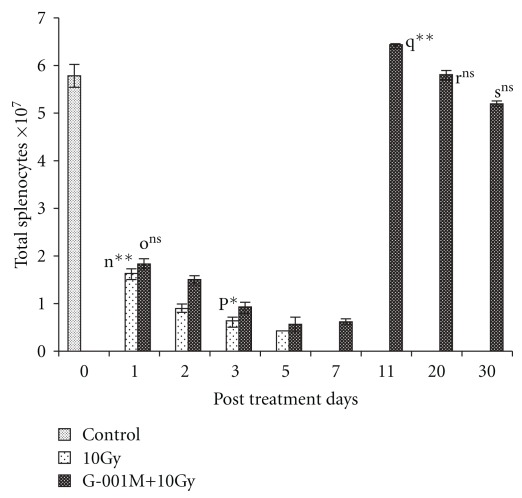
Effect of *P. hexandrum* sub-fraction (G-001M) on splenocytes of whole body irradiated (10 Gy) Swiss Albino mice. Animals were sacrificed at different time intervals. Experiments were done in triplicate with three animals in each group. n: Control versus 1st day 10 Gy; o: 1st day 10 Gy versus 1st day (G-001M + 10 Gy); p: 1st day 10 Gy versus 3rd day 10 Gy; q: 7th day (G-001M + l0 Gy) versus 11th day (G-001M + 10 Gy); r: Control versus 20th day (G-001M + 10 Gy); s: Control versus 30th day (G-001M + 10 Gy). (*P < .05, **P < .001, ns, non significant). All the values are reported as mean ± SD for three measurements.

Figure 9 depicts the number of splenocytes (×10^7^ cells/spleen) at different intervals of treatment with and without the administration of the formulation. In radiation only group, there was a drastic fall in spleen cell counts on the first day (5.78 ± 0.28 versus 1.62 ± 0.12, *P* < .001; control versus 10 Gy) of exposure, and the similar trend continued till 5th day (0.42 ± 0.008). Beyond the seventh day of exposure, mice in radiation only group rather showed more debris in their spleens than any surviving cell. In G-001M pre-treated irradiated groups also, the number of splenocytes declined sharply during the initial phase of study (up to 5th day), but replenished afterwards and on 11th day, the number of splenocytes (6.43 ± 0.078, *P* < .05) surpassed even the control values (Figure 9). On the 20th day, the total spleen cell count (5.8 ± 0.09, *P* = ns) in G-001M pre-treated irradiated group regained and matched with the control group. On later intervals of study, stability in splenocytes cell count was a regular observation.

### 3.7. Total Thymocytes Count

**Figure 10:**
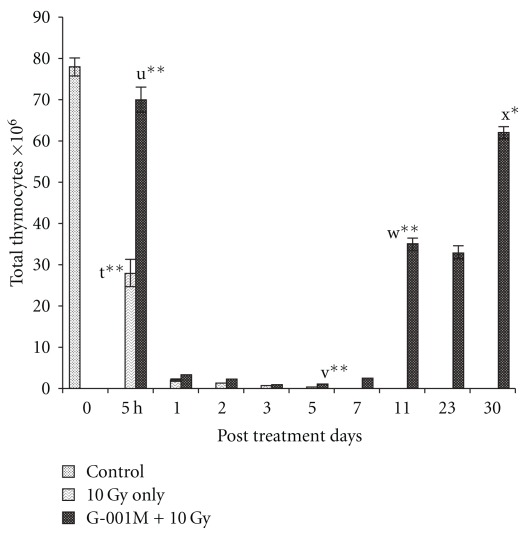
Effect of *P. hexandrum* sub-fraction (G-001M) on thymocytes of whole body irradiated (10 Gy) mice. Animals were sacrificed at different time intervals. Experiments were done in triplicate with three animals in each groups. t: Control versus 5 h 10 Gy; u: 5 h 10 Gy versus 5 h (G-001M + 10 Gy); v: 5 h 10 Gy versus 5th day 10 Gy; w: 7th day (G-001M + 10 Gy) versus 11th day (G-001M + 10 Gy); x: Normal versus 30th day (G-001M + l0 Gy). (*P < .05, **P < .001). All the values are reported as mean ± SD for three measurements.

In radiation only group, significant reduction in thymocytes count (×10^6^ cells/thymus) was registered at 5 h post irradiation (78 ± 2.1 versus 28 ± 3.2, *P* < .001, control versus irradiation), and the depletion progressed further, showing the counts drastically declined by 5th day (0.3 ± 0.02) of exposure as compared to control (Figure 10). Animals in the irradiated group showed fully regressed thymus in seven post irradiation days. However, with G-001M pre-treatment, though reduction in thymocytes count was recorded up to 5th day (1.1 ± 0.008), the fall was less prominent as compared to radiation only group. Thymocytes in this group started recovering from the 7th day (2.5 ± 0.05) onwards. The values improved splendidly by 11th day (35 ± 1.36) and reached to normal by the 30th day (62 ± 1.48, *P* = ns, control versus G-001M pre-treated irradiated).

## 4. Discussion

*Podophyllum hexandrum* derived crude and semi-purified extracts have been explored extensively for their radioprotective properties against lethal irradiation using *in vitro*, *ex vivo* and *in vivo* model systems [12–24]. Efforts were also made to understand underlying basic mechanism in rendering radioprotection [13, 15, 28, 29] by *P. hexandrum* formulations. Whole body survival, toxic doses and DRF were evaluated with two semi purified extracts (REC-2001 and REC-2006) of *P. hexandrum* [23, 24, 30, 31]. However, for further reduction in plant extract dose concentration and to minimize the toxicity, new formulations from this high altitude plant have been evaluated time and again. Sub-fraction G-001M, used in current study, is found superior to our earlier preparations in many aspects namely, effective through intramuscular administration, longer bioavailability of active principles, faster immune replenishment, reduced toxicity and protection to cellular DNA. Antioxidant potential of G-001M, checked using BHT as a standard, indicated that at particular concentration this formulation was able to reduce most Fe^3+^ ions (Figure 3), hence its reducing power was comparable to the standard used in the study. The reducing potential of G-001M is predominantly attributed to the presence of poly-phenols in significant quantity, which is in concordance with previous observations [15] too.

**Figure 11:**
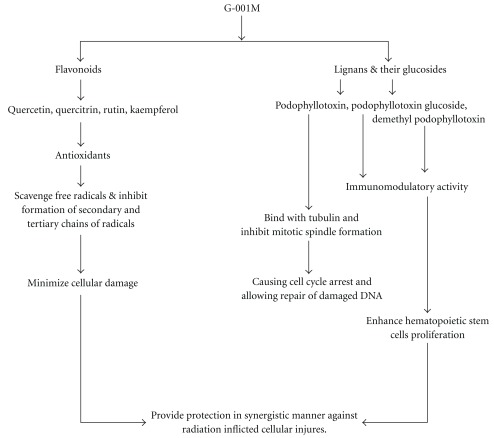
Hypothetical model to elucidate the mechanism of radioprotection of G-001M: a semipurified fraction of *P. hexandrum*.

HPLC of G-001M has confirmed the presence of podophyllotoxin, podophyllotoxin glucoside and demethyl podophyllotoxin (lignans) and flavonoids, namely quercetin, quercitrin, rutin and kaempferol. Both, phyto based lignans and flavonoids are well-known antioxidants, and their other pharmacological properties such as biocidal effects, anti carcinogenic, anti inflammatory and immunomodulatory have also been reviewed [9, 20, 22] intensely. Restoration of survival to the level of >90% against lethal radiation (0% survival) in mice (Figure 5) is apparently a result of multifaceted properties of G-001M working in synergism (Figure 11). The sustainability in % survival, from 30 min to 4 h, again indicates bioavailability of active principles of this sub-fraction in the body tissues for appreciably longer time and exhibiting significant survival during the entire period of retention. The formulation has not only been effective up to the lethal dose of radiation (10 Gy) but also 50% of the animals have survived against supra-lethal doses (13 Gy). The therapeutic dose (6 mg kg^−1^ body weight, i.m.) being very low from the toxic doses (MTD 80, LD_50_ 100, LD_100_ 120 mg kg^−1^ body weight.), is again a promising feature of G-001M.

Cellular DNA, the prime target of ionizing radiation, results into single and double strand breaks, base damage, cross linking and dimer formation which finally affects cell viability after exposure [1, 2]. ROS also interfere with normal cell signaling, regulation of transcriptional factors and modulation of protein kinase cascades [1, 2, 29]. Podophyllotoxin, a lignan, present in our sub-fraction, is well explored to have an additive role of DNA repair, primarily by G2-M cell-cycle block. This happens due to temporary inhibition in release of tubulin protein required for spindle formation to which each chromosome gets attached individually during the process of segregation [32]. Comet assay, used as an additional confirmatory test for evaluation of DNA protection and support to DNA repair rendered by our formulation, has shown significant reduction in radiation induced DNA tail length and DNA damage score in G-001M pre-treated group. These studies are in consonance with our previous study reported on mice thymocytes, wherein the other extract of *P. hexandrum* has also shown radiation induced thymocytic DNA protection [23, 24].

Glucosides of both podophyllotoxin and quercetin are known immunomodulators [33, 34]. The spurt in the number of thymocytes and splenocytes after a week of G-001M pre-treatment in radiation exposed mice, explicitly demonstrated that bone marrow precursor (stem cells) cells are not only well protected by our formulation but they also rejuvenated with the similar pace to maintain cellular immunity. Partially explained mechanism of action published earlier [29], exhibited by administration of this plant preparation before irradiation, has reflected up-regulation of certain proteins, such as PCNA (proliferating cell nuclear antigen) and Bcl_2_ (anti apoptotic protein), and down regulation of p53 (tumor suppressor protein) and Bax (pro-apoptotic proteins).

As well documented in other studies and confirmed by our studies too, support to immune system, hematopoietic system and cellular DNA [18, 20, 24] by most of radioprotective formulations from *P. hexandrum* is predominantly attributed to their properties of free radical scavenging, reduced lipid peroxidation [24], transient metal-chelation [35] and protection to endogenous defense enzymes [24] and to cellular DNA. The discontinuation of free radical mediated secondary and tertiary chain reactions has also greatly helped in minimization of multiplication of deleterious effects of ionizing radiation. Studies mentioned above have clearly demonstrated that G-001M has protected the animals by rendering multifaceted support to their biological system. The findings with *P. hexandrum* published so far were confined to intraperitoneal route of administration which is generally very cumbersome and painful, particularly, if to be used in humans. The latest prepared semi purified fraction (G-001M) has been studied exclusively with intramuscular route of administration to overcome drug administration related route apprehensions.

Finally, G-001M has been found holding enumerable properties, such as, simple extraction process, higher yield from comparatively less quantity of raw material, promising antioxidant potential, effective through intramuscular administration and wide gap in between therapeutic and toxic doses. This formulation has also rendered high degree of whole body survival, protection to hematopoietic and immune system, besides, assistance in DNA repair.

Plethora of significant findings reflected in current study strongly favor G-001M as a potential choice for developing a safe and effective formulation against lethal irradiation, resulting into human use with more intense studies.

## Figures and Tables

**Table 1:** (Lignans). High performance liquid chromatography (HPLC) of the bioactive fraction was performed using methanol:water gradient over a period of 70 min: column; RP-18 (E. Merck, 5 *μ*m, 4.0 × 250 mm); Flow rate 0.6 ml min^−1^; UV 290 nm.

Time (min)	Percentage of A (water)	Percentage of B (methanol)
00.01	65.00	35.00
60.00	35.00	65.00
70.00	65.00	35.00

**Table 2:** (Flavonoids). HPLC of the bioactive fraction was performed using acetonitrile containing 0.05% of tri-flouro acetic acid (TFA):water containing 0.05% of TFA gradient over a period of 70 min: column; RP-18 (E. Merck, 5 *μ*m, 4.0 × 250 mm); Flow rate 1.0 ml min^−1^; UV 250 nm.

Time (min)	Percentage of A (water)	Percentage of B (acetonitrile)
00.01	88.00	12.00
25.00	79.00	21.00
30.00	75.00	25.00
35.00	25.00	75.00
60.00	00.00	100.00
70.00	88.00	12.00
